# Usefulness of a biomarker to identify placental dysfunction in the context of malaria

**DOI:** 10.1186/s12936-016-1664-0

**Published:** 2017-01-03

**Authors:** Alexandra Gueneuc, Philippe Deloron, Gwladys I. Bertin

**Affiliations:** 1 Institute of Research for Development (IRD), UMR216-MERIT, Paris, France; 2 ComUE Sorbonne Paris Cité, Paris, France; 3 DHU Risks in Pregnancy, Paris, France; 4 Obstetrics and Fetal Medicine Department, Necker-Enfants-Malades Hospital, Paris, France

**Keywords:** Biomarker, Pregnancy malaria, Preeclampsia

## Abstract

In most tropical areas, pregnant women are at increased risk of malaria, as a consequence of the massive sequestration of parasitized red blood cells in the placenta. The placenta plays a key role in embryonic and fetal development as well as in maternal-fetal exchanges, and pregnancy-associated malaria may alter selected placenta functions that lead to stillbirth and low birth weight. Although there are several tools (blood smear examination, RDT, PCR) to diagnose malaria infection during pregnancy, there is currently no test to assess placenta dysfunction in the framework of pregnancy-associated malaria. Pregnancy-associated malaria shares many features with preeclampsia, an extensively studied disease. Various biomarkers associated with placental dysfunction have been identified as associated with preeclampsia. Several of these are inflammatory markers that lack of specificity. A few seem more specific of placenta dysfunction, including s-endoglin and sFlt1, increased in the peripheral blood during preeclampsia. The predictive value of these biomarkers should be studied in the context of pregnancy-associated malaria to evaluate their usefulness in identifying placental dysfunction during malaria. These biomarkers should be considered to improve the diagnosis of placental dysfunction during malaria and pregnant women monitoring.

## Background

### Placenta and syncytiotrophoblast cells

Placenta is a new transitional organ essential to the preservation of pregnancy and fetal development. It ensures maternal-fetal exchanges and is also involved in maternal tolerance of feto-paternal antigens. In some species, including human, the placenta exerts hormonal functions that are required for the maintenance of gestation and fetal well-being.

During pregnancy, a vascular shunt is formed, and maternal blood crosses the myometrium via utero-placental arteries to invade the intervillous space under maternal blood pressure. Throughout pregnancy, syncytiotrophoblasts (ST) evolves towards cellular death by apoptosis in the intervillous space. Their renewal is a continuous phenomenon, and is more pronounced in case of placental suffering. ST represent the first mother-fetal interface, and fulfil many functions. They also have a major endocrine role by participating in the steroidogenesis and in the synthesis of pregnancy-specific hormones, human chorionic gonadotrophin (hCG) and human placental lactogen (hPL). hCG is a placentation early marker, and a functionality marker [1, 2]. Finally, the ST have an immuno-modulation function by allowing maternal tolerance in front of the semi-allogenic transplant of the feto-placental unit.

### Malaria infection

*Plasmodium falciparum* infection is contracted from an anopheles bite by injection of sporozoites, which quickly begin an intra-hepatocytic maturation phase. Merozoites are released from the hepatocyte and invade erythrocytes, beginning a 48-h long erythrocytic cycle during which the parasite goes through several morphological stages, from ring to trophozoite and schizont [3]. The key step during this erythrocytic cycle is the important trafficking of proteins. Indeed, *P. falciparum*-derived proteins are successively expressed, some are exported, and presented at the surface of infected erythrocytes (iE) cell membrane, leading to physical and morphological alterations of iE. iE shape and deformability are modified, and electron dense protrusions, the knobs, appear at their surface. Knob structure involves complex interactions between parasite proteins and the iE membrane cytoskeleton. Knobs are involved in the export of variant surface antigens (VSAs), such as *P. falciparum* erythrocyte membrane protein-1 (*Pf*EMP-1), mediating the adhesion of iE to endothelial cell receptors, a phenomenon required for the completion of the erythrocytic cycle. *Pf*EMP-1 is a highly variable protein, allowing the parasite to escape host immunity, and to specifically adhere to endothelial cells or to deep organs, such as the surface of the ST at the intervillous spaces of the placenta. This adhesion requires a specific receptor on the endothelial cell, which in the case of the ST is chondroitin sulphate A (CSA) [4]. By presenting these ligands, the parasite allows host immune recognition of the altered iE surface. To evade the immune response, these proteins display extensive antigenic variation, concurrently changing the receptor recognition, and then the tissue tropism of the iE [5]. One member of the *Pf*EMP-1 family, VAR2CSA, is specific of parasites encountered during pregnancy. Only the parasites expressing the VAR2CSA variant are able to sequester in the placenta and those parasites cannot bind to receptors expressed elsewhere, therefore, they have never been able to multiply in the host before the first pregnancy, and no immune response against VAR2CSA exists [6]. The binding is strengthened by the attraction of their opposite charges and resists the maternal blood flow [7]. The result is the adherence and accumulation of iE in the intervillous space. This parasite adhesion to placental receptor CSA is the initiator of the immune response to CSA-binding iE, fundamental to the pregnancy-associated malaria (PAM) pathogenesis.

### Pregnancy-associated malaria

The sequestration of numerous iE in the placenta is responsible for inflammatory damages with fibrinoid deposits at its surface, similar to chronic intervillitis [8]. These damages may alter the placenta functions and lead to delayed fetal growth. Malaria infection has pro-inflammatory effects, in particular on infected placentas where appear an oxidative stress and fibrin deposits. iE sequestration leads to acute vascular modifications of placental villi, reducing the available surface for feto-placental exchanges. This decrease may be correlated with intra-uterine growth retardation [9, 10]. The blood flow modifications by the accumulation of iEs in the intervillous spaces originate ST hypoxia and angiogenesis deregulation. The long-term placental infringements may result in low birth weight, in utero fetal death, and important perinatal morbi-mortality [11]. Abnormal intra-uterine environment (exchange defect and hypoxemia) affects the cognitive, metabolic and anthropometric development of the fetus, which may lead to a greater risk of disease for the newborn child [12]. In *P. falciparum*-infected pregnant women, ultrasound studies reported intra-uterine fetal growth restriction, and Doppler analysis showed fetal brain vasodilatation, witness of hypoxia [13].

### Current recommendations for PAM diagnosis and prevention

For pregnant women living in moderate to high malaria transmission areas, the World Heath Organization (WHO) recommends the use of long-lasting insecticide-impregnated bed nets and intermittent preventive treatment (IPT) which consists of a curative regimen of sulfadoxine-pyrimethamine, from the second half of pregnancy, during each antenatal visit providing those are spaced by at least one month. This prevention allows to reduce the number of malaria attacks during pregnancy, the placental sequestration, and the fetal consequences [14]. Nevertheless, IPT is only partially effective in preventing malaria infection during pregnancy and its consequences, due to several reasons, including the lack of coverage of the first trimester by IPT, and the high prevalence of parasite resistance to sulfadoxine-pyrimethamine in most malaria endemic areas.

Women living in areas of stable malaria present even (or non-existent) symptoms that do not allow predicting placental infringement. In clinical practice, the biological diagnosis of malaria relies on thick blood smear microscopic examination and rapid diagnostic test (RDT). The thick smear allows to estimate the parasite density in the maternal peripheral blood, but does not reveal the level of placental parasite sequestration. RDT may be a better diagnostic tool for use in pregnant women, as *P. falciparum* sequesters in the placenta and, therefore, may not be present in the peripheral blood, producing false-negative results of the blood smear. For the WHO, the RDT is now the reference test [15]. However, the evidence of the presence of parasites in peripheral blood (either by blood smear or RDT) does not inform the clinician about the importance of placental sequestration, nor about the current placental function. In addition, both thick blood smear examination and RDTs suffer from a lack of sensitivity, and the molecular diagnosis by PCR allows to evidence as many as 2–4 times more women infected by *P. falciparum* experiencing a so-called sub-microscopic infection. These sub-microscopic infections are also associated with poor pregnancy outcomes, as maternal anaemia, premature birth and low birth weight are more frequent [16]. Currently, no tool is available in endemic areas to identify suffering placentas. Such a tool would be of great interest, allowing to identify patients at risk of PAM complications, such as IUGR. A curative treatment could potentially be initiated, and an observation of the fetus suffering from the defective functions of the placenta should be initiated.

### Immunology during pregnancy and PAM

An immunological balance during pregnancy allows the tolerance of the semi-allogenic transplant, represented by the feto-placental unit. This balance contains three successive immunological phases. The first is a strong inflammatory response during the implantation, the placentation and the first quarter of pregnancy. Then, arises an anti-inflammatory phase, with a Th2 type strong response to assure the mother-fetus symbiosis and fetus development. Pregnancy maintenance is dependent on this cytokine profile with CD4^+^ cells secreting IL-4, IL-5, IL-6, IL-10, and IL-13 cytokines [17]. Finally, the parturition requires a strong pro-inflammatory response to induce the uterine contractions. Th1 lymphocytes produce mainly IL-12, IFN-γ, and TNF-β that stimulate the production of prostaglandins and metalloproteases, leading to delivery [18]. This balance of the Th1/Th2 responses is essential to pregnancy, but leads pregnant woman more susceptible to infections, including malaria.

Adult women living in malaria endemic areas have acquired a protective immunity to the repertoire of the *P. falciparum* variants they have been previously infected with. During pregnancy, their susceptibility to malaria infection increases. In areas of stable malaria transmission, this increase in susceptibility is parity-dependent, being highest in primigravidae. Several hypotheses have been suggested to explain this. Pregnant women would be more attractive to *Anopheles* and thus more bitten by mosquitoes than non-pregnant women [19]. Increased susceptibility has also been explained by the immunomodulation related to the Th1/Th2 immune response imbalance. The increase of plasma factors, such as cortisol and prolactine, can inhibit the inflammatory response necessary for the control of the parasites [18]. However, none of these observations explain the higher prevalence of malaria in primigravidae, nor the elective localization of iE in the placenta [20]. The identification of VAR2CSA variants allowed emitting a new hypothesis. The emergence of a pregnancy-specific variant corresponds to the parasite adaptation in its ability to bind CSA, a new receptor present at the surface of the ST of the placenta. The lack of previously—acquired specific immunity for this new variant antigen allows the development of the infection [6].

Epidemiological and immunological features of malaria infection during early pregnancy are almost completely unknown, as in most malaria endemic countries women first attend to maternity wards at a late gestational age, usually in the second half of second trimester. At the placental level, IE binding promotes intense infiltration of immune cells [21, 22] in the intervillous spaces, resulting in an important pro-inflammatory cytokines production necessary to parasite elimination, but harmful to pregnancy. The placental inflammation promotes placental lesions and exchanges imbalance that are deleterious for intrauterine fetal growth [11, 23]. Disruption of angiogenesis through elevated levels of sFlt-1 (or sVEFGR1) in the second trimester has been also reported [24]. The building of the vascular network is mediated by the VEGF protein, a pro-angiogenic factor expressed by trophoblast that can bind to its receptor, sFlt-1, and inhibit its activity. The consequences of this local inflammation are maternal anemia, in utero growth retardation and trans-placental transfer of soluble antigens to the fetus [23]. ST shapes local immunity by recruiting mononuclear cells from the peripheral blood. Immune balance aims towards Th1 cytokines, such as TNF and IFN-γ. Yet, in primigravidae, TNF-α and IFN-γ increase correlates with severe anaemia and low birth weight [25]. Activated macrophages produce IL-1, IL-6, TNF and free radicals that engender placental changes, and disturb the mother-fetal exchanges [26]. To protect ST, anti-inflammatory cytokines, such as IL-10, are increased [27].

In the peripheral blood, high concentrations of plasmatic IL-10, TNF and G-CSF induce an inflammatory response [28]. In asymptomatically infected pregnant women, parasite density is correlated to the levels of soluble mediators of inflammation, such as soluble TNF receptors (TNF-R1 and TNF-R2), IL-10 and G-CSF, suggesting these factors may represent markers of PAM-related inflammation [28, 29]. Altered DCs and monocytes, and reduced frequency and low expression of the CD86 and CD80 activation markers, are observed in malaria-infected pregnant women, suggesting DC migration to lymphoid organs [30]. Hemozoin, the malaria pigment, may induce DC activation and maturation via the expression of CD80, CD86 and chemokine receptors CXCR4, promoting their migration to lymphoid organs [31].

The development of specific humoral immunity to VAR2CSA tries to limit the influx of inflammatory cells. During successive pregnancies, women develop specific immune memory against VAR2CSA variants, and acquire protective immunity. Multigravidae possess antibodies against VAR2CSA that efficiently inhibit the adhesion of iE to ST. The presence of anti-VAR2CSA antibodies at a high titre from early pregnancy until term allows to protect women from placental infection [32]. Antibodies against VAR2CSA are essential in the elimination of the parasites, and the level of antibodies allows to estimate the level of immune protection against placental malaria [33]. Primigravidae exposed to *P. falciparum* are able, from the first quarter of pregnancy, to produce these specific antibodies, but to a low level, insufficient to confer effective protection against malaria [34].

Currently, numerous biomarkers, mainly inflammatory markers, have been studied in the context of PAM. A systematic review of the topic reported that all these biomarkers were unspecific and none shown a significant advantage as single biomarker [35]. However, a combination of multiple biomarkers involved in different pathophysiological pathways of PAM may increase specificity, and could have the needed diagnostic value to identify at-risk patients. Other pathologies leading to fetal growth restriction have been much more extensively studied than PAM. In these, biomarkers indicative of placenta dysfunction have been identified and may be of interest in the context of PAM. A biomarker of placenta dysfunction, even if not specific to malaria, but associated with a positive thick smear or RDT could determine the women most at risk of complications. One of the most extensively studied among such pathologies is preeclampsia that shares numerous pathophysiological features with PAM.

### Similarities between PAM and preeclampsia

Preeclampsia is due to a placental dysfunction that releases in the maternal circulation substances (free radicals) causing an endothelium infringement. There are epidemiologic similarities between PAM and preeclampsia, both being more frequent in primigravidae [36], and a major cause of maternal mortality in developing countries [36, 37]. Some authors described similar seasonal changes in the incidence of both preeclampsia and PAM, with increased incidence during the rainy season [38, 39]. Clinically, reduced placental perfusion is a common link between these pathologies that are associated with fetal growth restriction [10, 40]. Indeed, preeclampsia translates into a high arterial blood pressure, a glomerular renal disease with proteinuria, and can be complicated by lung edema, renal insufficiency, and eclampsia. Placental dysfunction is the consequence of an immunological imbalance that originates a deficit of the vascular reshaping of maternal arteries during the trophoblastic invasion by extravillous cytotrophoblasts (EVCT). An excessive activation of the maternal immune response impairs the recognition of trophoblastic cells and their lysis. The defect of placental vascularization causes a decrease of blood flow in the intervillous spaces, gradually leading to chronic hypoxia. The oxidative stress increases apoptotic fragments of the ST, and different factors (free radicals, oxidized lipids, cytokines, sFlt-1, s-Endoglin) are released in the maternal circulation where they damage the endothelium. During preeclampsia, sFlt-1 levels are increased, and VEGF levels are significantly decreased [41]. Several reports suggest that an inflammatory reaction, and an abnormal secretion of secondary cytokines during placental malaria might be initiators of severe preeclampsia and eclampsia [42–44].

Several preeclampsia biomarkers have been described to detect patients at risk. sFlt-1, the soluble receptor of VEGF having an anti-angiogenic effect, is increased in the peripheral blood, and its level correlated with the severity of the preeclampsia syndrome [45]. High concentrations of sFlt-1 disturb angiogenesis, even worsened by elevated maternal blood levels of s-Endoglin. This trans-membrane anti-angiogenic glycoprotein is also increased in the maternal blood in the second quarter of pregnancy [46]. Levine et al. [47] reported a mean sFlt-1 value three times higher in women with preeclampsia than in normal pregnancies. Women with subsequent preeclampsia had twice higher concentrations of sFlt-1 at 16–20 weeks than the controls [24]. Placental protein 13 (PP13) is also increased in preeclampsia [48], whereas Pregnancy-associated plasma protein A (PAPP-A) glycoprotein synthesized by the placenta is decreased. The placenta is also a source of circulating inflammatory cytokines, chemokines or immunoglobulins like IFN-γ, MCP-1 and ICAM-1, VCAM-1 [49], as well as TNF in the third quarter of pregnancy are increased in patients with preeclampsia [50]. Inhibin A, involved in gonadotropin repression, is also increased in preeclampsia. Conversely, the level of visfatin, a placental factor involved in glucose homeostasis, is decreased in the maternal blood [45].

Several molecules are increased in plasma during PAM, including ferritin with anti-inflammatory functions, as well as leptin and IFN-γ. An increase of IL10, that has an anti-inflammatory role and a negative feedback on TNF, has specificity and sensibility of placental inflammation in PAM [51]. The immune response to CSA-binding iE involves TNF and its receptors TNF-R1 and R2. They stimulate the phagocytic activity of leukocytes by increasing ICAM-1 expression and the pro-inflammatory response by favouring the liberation of IL6 and IL8 [51]. Other biomarkers have been described as increased in the peripheral blood during PAM: IL1, IL10, TNF, TNF-R1, TNF-R2 and IFN-γ [52, 53].

Similarities between PAM and preeclampsia are striking and could lead to define biomarkers identified within the framework of preeclampsia as PAM’s potential markers. Indeed, several markers are increased in both pathologies, including TNF, IFN-γ, ICAM-1, IL10, and IL6, but those are lacking specificity. Other factors, such as nitric oxide synthase, s-endoglin, or TGF-ß soluble receptor are associated to preeclampsia, but also to severe malaria in children and in infected primigravidae [44]. This oxidative stress has been reported in several placental disorders, pregnancy pathologies, and also in PAM [54, 55]. Malondialdehyde (MDA) is correlated positively with parasitaemia and NO is correlated negatively with birth weight [55]. sFlt-1 is also increased during PAM and associated with fetal loss, intra-uterine growth restriction, and placental inflammation.

Duffy’s study reported a correlation between PAM and hypertension in young primigravidae. In this population, levels of sFlt-1 are elevated, suggesting its involvement in this correlation [42]. These similarities between malaria infection and preeclampsia open additional horizons. All these biomarkers are thus potential candidates for the diagnosis of PAM with placental damage, but have not been validated in clinical practice. A randomized study may allow validating their interest to improve the detection of a suffering placenta as a consequence of malaria infection.

Preeclampsia screening involves monthly blood pressure and proteinuria check. The diagnosis is made after 20 gestational weeks if the blood pressure is more than 14/10 mm/hg and proteinuria >0.3 g/24 h. Actually, new ways of detection are studied, adding to these measures the uterine Doppler and the PlGF and sFlt-1 dosages. Combined they should identify, in the third trimester, 84% of preeclampsia with 10% of false positive [56]. Indeed, the biomarkers can help to the screening of women at risk of preeclampsia. But this has to be confirmed. In France, numerous laboratories work on this hypothesis that is not currently included as standard practice, only blood pressure and urine protein test being part of the monitoring during prenatal consultations. Moreover, there are suspicions (although this is not clearly demonstrated) that malaria during pregnancy is a risk factor for preeclampsia, most probably through placental alterations. In a malaria-endemic area, it is certainly difficult to manage preeclampsia, but women presenting an sFlt-1/PlGF high level should be submitted to protein urinary stick and blood pressure measurement.

## Conclusion

**Fig. 1:**
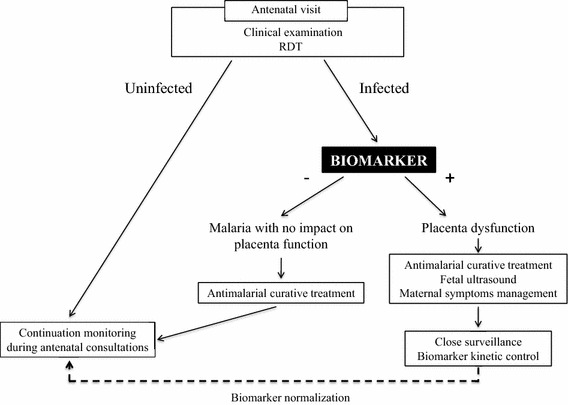
How a biomarker of placental dysfunction can help to identify women at greater risk of poor pregnancy outcome?

Identification of patients at risk of poor pregnancy outcome thanks to biomarkers will allow to optimize the prevention of the consequences of placental sequestration. Currently, the management of PAM, by long-lasting insecticide-impregnated nets (LLIN) and intermittent preventive treatment in pregnancy, occurs often late, and is taking care of malaria infection but not of its consequences on the placenta that may persist after having cure the infection. It seems clear that the availability of a tool to identify, at an early stage, a placenta ‘under stress’ resulting from PAM would represent an important step forward in terms of diagnosing and treating one of the main causes of poor pregnancy outcomes in African populations. Such biomarker would help in understanding the relationships between the timing and severity of *P. falciparum* infections and poor pregnancy outcomes, and to optimize preventive and curative interventions. A biomarker of placenta dysfunction along with a positive thick smear or RDT, should evidence a suffering placental. This screening test would allow to add to IPT a regular monitoring during the pregnancy with a more appropriated treatment. It would be a primary tool to predict poor pregnancy outcomes, to adapt monitoring, and to ensure a close clinical and sonographic surveillance of these patients (Fig. 1). PAM increases the risk of preterm delivery by the secretion of cytokines, and the risks of IUGR by chronic hypoxia of the fetus. The identification of women at risk would allow the initiation of curative treatment during the pregnancy by artemisinin-based combination therapy (ACT) [57]. If the patients have severe malaria, treatment with artesunate IV is recommended in complement with paracetamol to limit the hyperthermia. In case of severe anaemia (Hb <7 g/dl), blood transfusion will be needed [57]. An echography to estimate the fetal weight, with Doppler measure could be realised at the beginning of treatment, and at 15 days intervals. The Doppler will allow to evaluate the level of placental alterations. Indeed, the Doppler allows, in function of the gestational age, to evaluate the benefice to follow-up the pregnancy in case of severe IUGR [58, 59]. Additionally, variations in the biomarker level could have a prognostic value for the surveillance of placenta stress evolution. Of course, these prerogatives cannot be currently implemented in all health centres in malaria transmission areas. However, things are moving fast, and ultrasound apparatus are now available in an increasing number of health centres in tropical areas. The goal is also to improve the surveillance of pregnancies, access to screening, to treatments, and the evaluation of the fetal health by Doppler echography.

## Abbreviations

CSA: chondroitin sulphate A
EVCT: extra villous cytotrophoblast
hCG: human chorionic gonadotrophin
hPL: human placental lactogen
iE: infected erythrocytes
IPT: intermittent preventive treatment
IPTp: intermittent preventive treatment in pregnancy
IUGR: intrauterine growth restriction
LLIN: long lasting insecticidal nets
*Pf*EMP-1: *Plasmodium falciparum* erythrocyte membrane protein-1
PP13: placental protein 13
PAM: pregnancy-associated malaria
PAPP-A: pregnancy-associated plasma protein A
RDT: rapid diagnostic test
ST: syncytiotrophoblast
VSAs: variant surface antigens
WHO: World Heath Organization
